# Staphylococcus capitis Sepsis Complicated by Rapid Native Aortic Valve Endocarditis in Sickle Cell Disease: A Case Report

**DOI:** 10.7759/cureus.104854

**Published:** 2026-03-08

**Authors:** Rohan M Patel, Tiffany S Sculthorpe, Alisha Abad, Kashif Abad

**Affiliations:** 1 College of Medicine, Nova Southeastern University Dr. Kiran C. Patel College of Osteopathic Medicine, Fort Lauderdale, USA; 2 Internal Medicine, Broward Health, Fort Lauderdale, USA; 3 Patient Care, Broward Health, Fort Lauderdale, USA

**Keywords:** cardiothoracic & vascular surgery, infectious disease medicine, infective endocarditis, native aortic valve endocarditis, sepsis, sickle cell disease complications, staphylococcus capitis, surgical aortic valve replacement (savr), transthoracic echocardiogram

## Abstract

Infective endocarditis involving the aortic valve carries a high risk of rapid valvular destruction and hemodynamic compromise, particularly when complicated by sepsis. Patients with sickle cell disease are uniquely susceptible to severe infection due to functional asplenia, chronic hemolysis, endothelial dysfunction, and frequent exposure to intravascular devices, yet native valve endocarditis in this population remains rarely described. We report the case of a 51-year-old man with sickle cell disease who presented with sepsis and was found to have native aortic valve endocarditis caused by *Staphylococcus capitis*. Initial transthoracic echocardiography on hospital day three demonstrated a small aortic valve vegetation with mild-to-moderate aortic regurgitation. Despite initiation of appropriate antimicrobial therapy, transesophageal echocardiography performed thereafter on hospital day six revealed rapid progression to moderate-to-severe aortic regurgitation with associated atrial dilation, indicating accelerated valvular deterioration. The patient subsequently developed worsening volume overload and hemodynamic instability, prompting cardiothoracic surgical intervention. He underwent successful aortic valve replacement with perioperative transfusion support tailored to the hematologic and hypoxic risks associated with sickle cell disease and demonstrated clinical improvement postoperatively. This case highlights how sepsis-related inflammatory activation, superimposed on baseline vascular and hematologic abnormalities in sickle cell disease, may accelerate valvular destruction, even in infections caused by organisms traditionally considered low-virulence, underscoring the importance of serial echocardiographic assessment and timely multidisciplinary management.

## Introduction

Infective endocarditis (IE) is an infection of the cardiac endocardium, with a predilection for valves. Aortic valve involvement is associated with abrupt valvular destruction and hemodynamic compromise if not addressed promptly [1]. Common etiological pathogens include *Staphylococcal*, *Streptococcal*, and *Enterococcal* species; indwelling intravascular devices and ports are recognized as a prominent portal of entry [2]. IE continues to carry a significant risk, especially when clinical presentations are complicated by sepsis; this is secondary to amplified systemic inflammation, higher embolic burden, persistent bacteremia from the vegetations, and limited tolerance for surgical intervention [3].

Patients with sickle cell disease (SCD) are particularly vulnerable to invasive infections secondary to functional asplenia, chronic hemolysis, and repeated vascular access for transfusions [4]. Sepsis in SCD is further complicated by microvascular occlusion, heightened inflammatory response, and baseline cardiovascular stress. This is because sepsis exacerbates hypoxia and systemic inflammation, promoting erythrocyte sickling and endothelial adhesion that propagate microvascular occlusion. Simultaneously, chronic anemia and a baseline high-output cardiac state may worsen susceptibility to cardiac collapse, secondary to heart failure, endothelial dysfunction, or increasing ventricular wall stress [5]. *Staphylococcus capitis,* although a low-virulence coagulase-negative *Staphylococcus*, has increasingly been recognized as a pathogen associated with device-related infections and prosthetic valve endocarditis [6]. Native valve infection in the setting of SCD, however, remains sparsely reported. 

This case describes native aortic valve IE in an adult with SCD resulting in moderate-to-severe aortic regurgitation and hemodynamic instability requiring valve replacement. This hospital course highlights the synergistic effects of sepsis, endothelial dysfunction, and active bacteremia in promoting rapid valvular deterioration, while recognizing the complexity of balancing anticoagulation, neuroprotection for stroke and cerebral anemia prevention, and antibiotic stewardship in SCD.

## Case presentation

**Figure 1:**
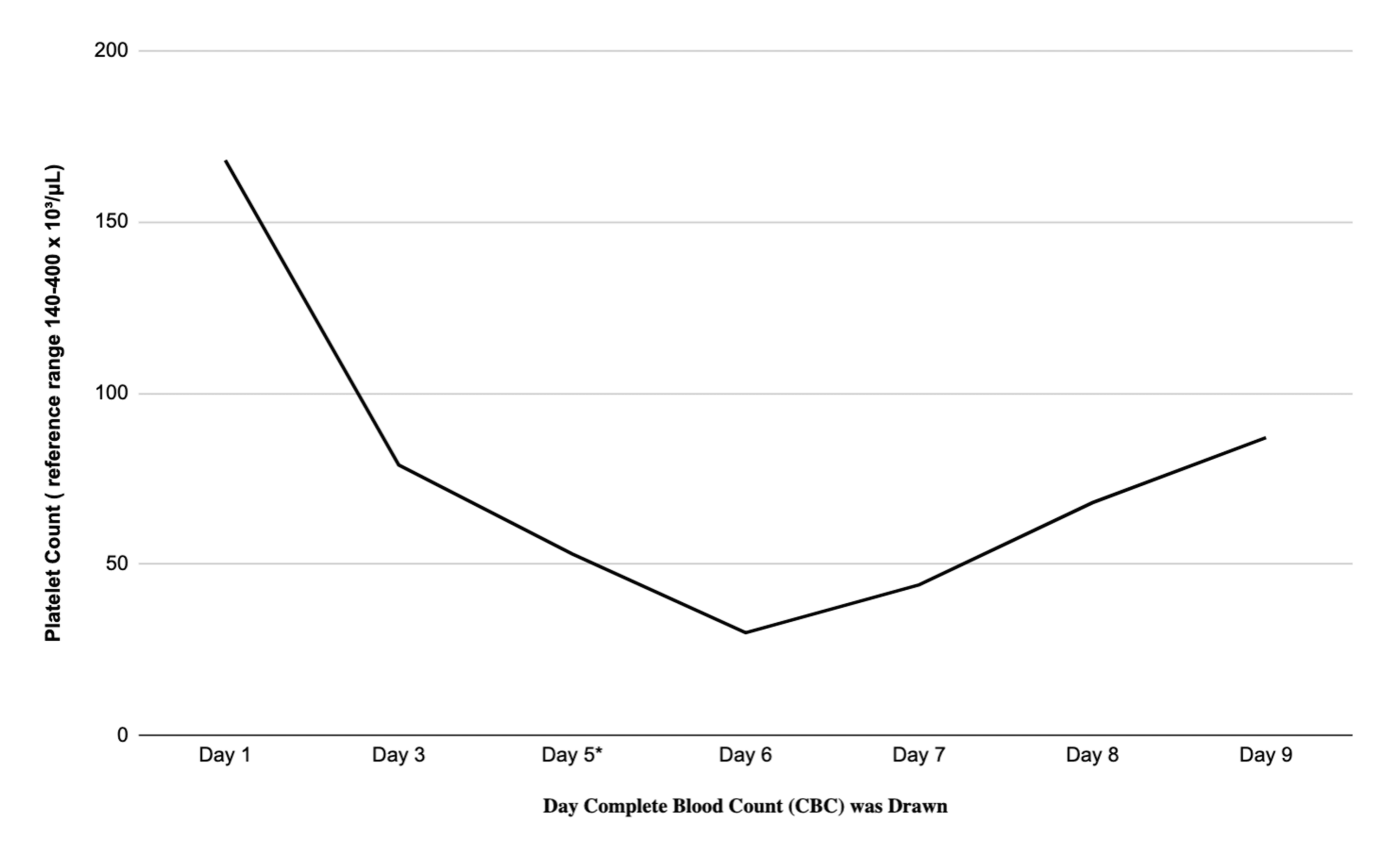
Serial platelet counts demonstrating thrombocytopenia during cefazolin therapy with progressive recovery following drug discontinuation. *Cefazolin was discontinued on day five due to progressive thrombocytopenia, and vancomycin was reinitiated. Note: Days represent the relative timing of CBC measurements during hospitalization.

A 51-year-old male with known SCD, a chest venous access port insertion for transfusion in 2021, multiple hospitalizations for sickle cell pain crises, and chronic lower extremity ulcerations, presented to the emergency department with five days of fever, chills, and a non-productive cough. Initial evaluation revealed a temperature of 102.2°F, sinus tachycardia at 125 bpm, and a leukocytosis of 16.5 × 10³/μL (reference range 4.00-11.00 10³/μL). Piperacillin-tazobactam (3.375 g IV every eight hours) and vancomycin (750 mg IV every 12 hours) were empirically initiated. Two blood cultures returned positive for gram-positive cocci in clusters, later identified as *Staphylococcus capitis. *The susceptibility report shortly returned, which depicted the pathogen as being oxacillin-sensitive. Antibiotics were then narrowed to cefazolin by the infectious disease team; however, worsening thrombocytopenia (Figure 1) prompted a transition back to vancomycin. The rapid drop in platelet count, coupled with no other clinical signs pointing to an alternate diagnosis, prompted antibiotic transition due to suspected beta-lactam-induced thrombocytopenia.

**Figure 2:**
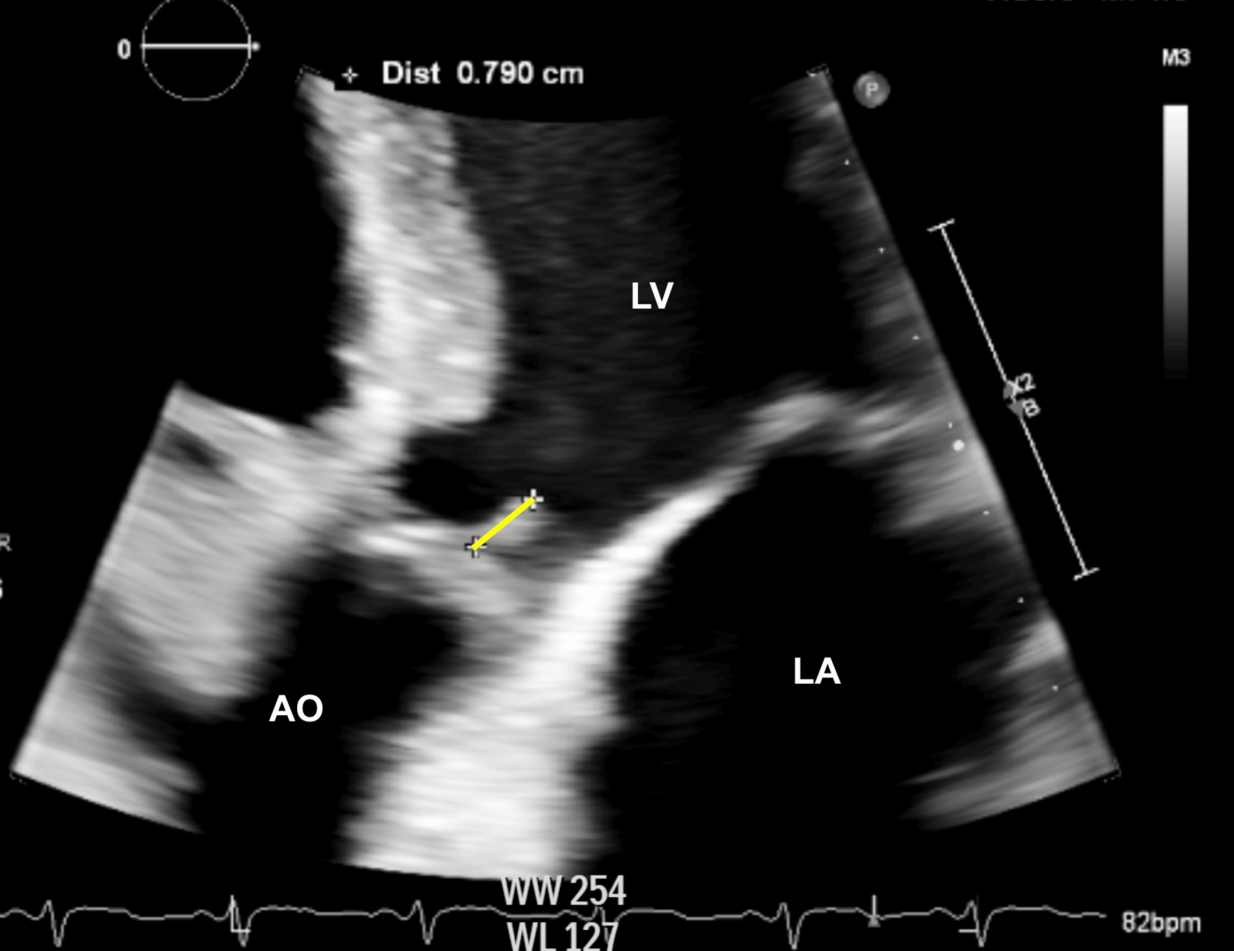
Transthoracic echocardiogram (TTE) displaying 0.79 cm vegetation on the aortic valve (yellow line). LA: left atrium; LV: left ventricle; AO: aorta.

Before admission, the patient had a structurally normal heart with normal function, confirmed by an echocardiogram four years prior, depicting no valvular dysfunction and an ejection fraction of 60% (the threshold for transfusion was a hemoglobin level <8.0 g/dL (reference range 13.0-17.3 g/dL)). Transthoracic echocardiography (TTE) on hospital day three revealed 0.79 cm vegetation on the aortic valve with mild-to-moderate regurgitation, raising an initial concern for aortic valve IE (Figure 2). At this time, the infectious disease team recommended a follow-up transesophageal echocardiogram (TEE) to better assess the vegetation. Repeat blood cultures returned at this time and were negative. Cardiothoracic surgery was also consulted for venous port removal, which was scheduled to be completed after the TEE was performed.

**Figure 3:**
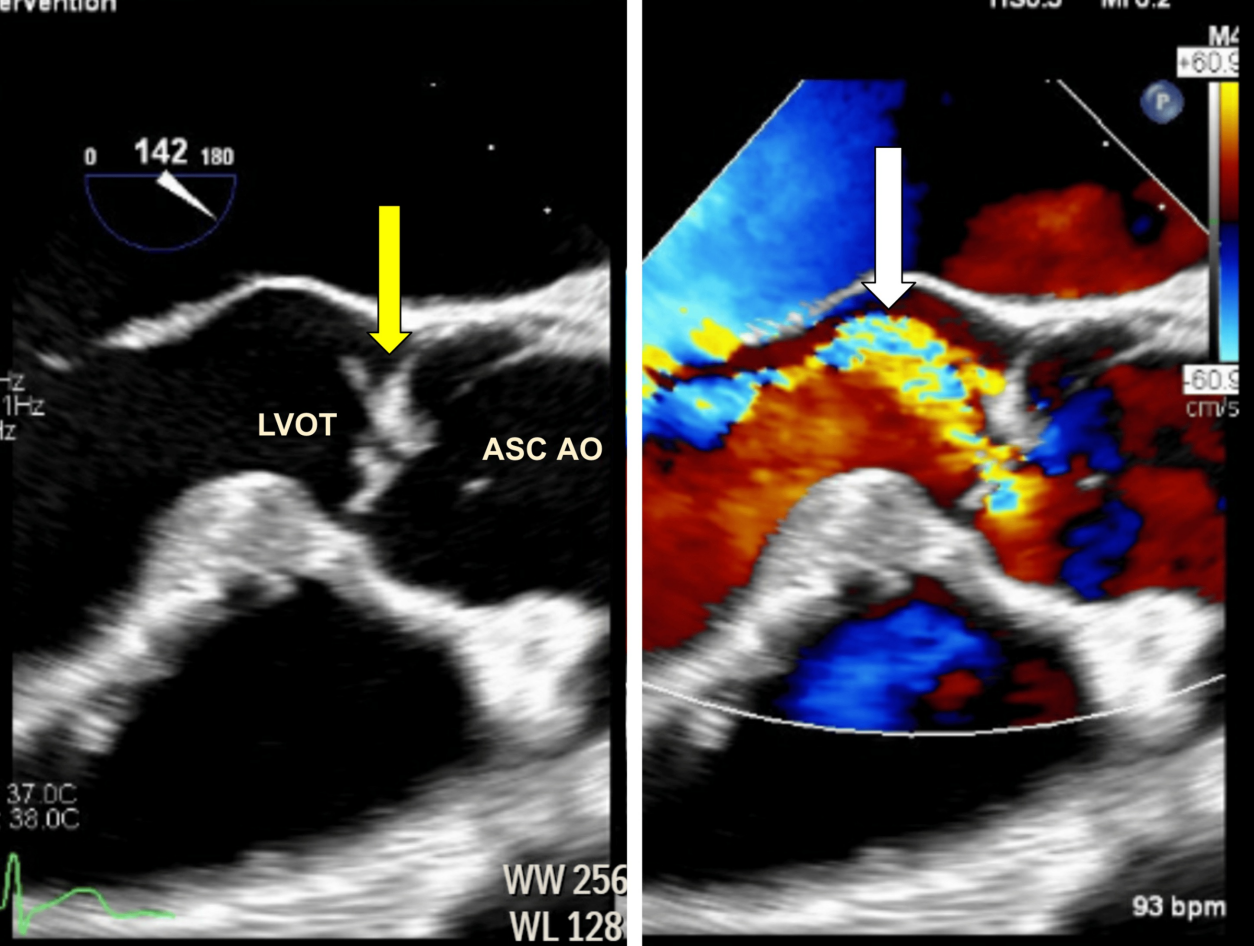
Transesophageal echocardiogram (left) depicting an echogenic area involving the right and left coronary cusp, consistent with endocarditis (yellow arrow). Color flow study (right) revealing moderate-to-severe aortic regurgitation secondary to turbulent flow (white arrow). LVOT: left ventricular outflow tract; ASC AO: ascending aorta.

**Figure 4:**
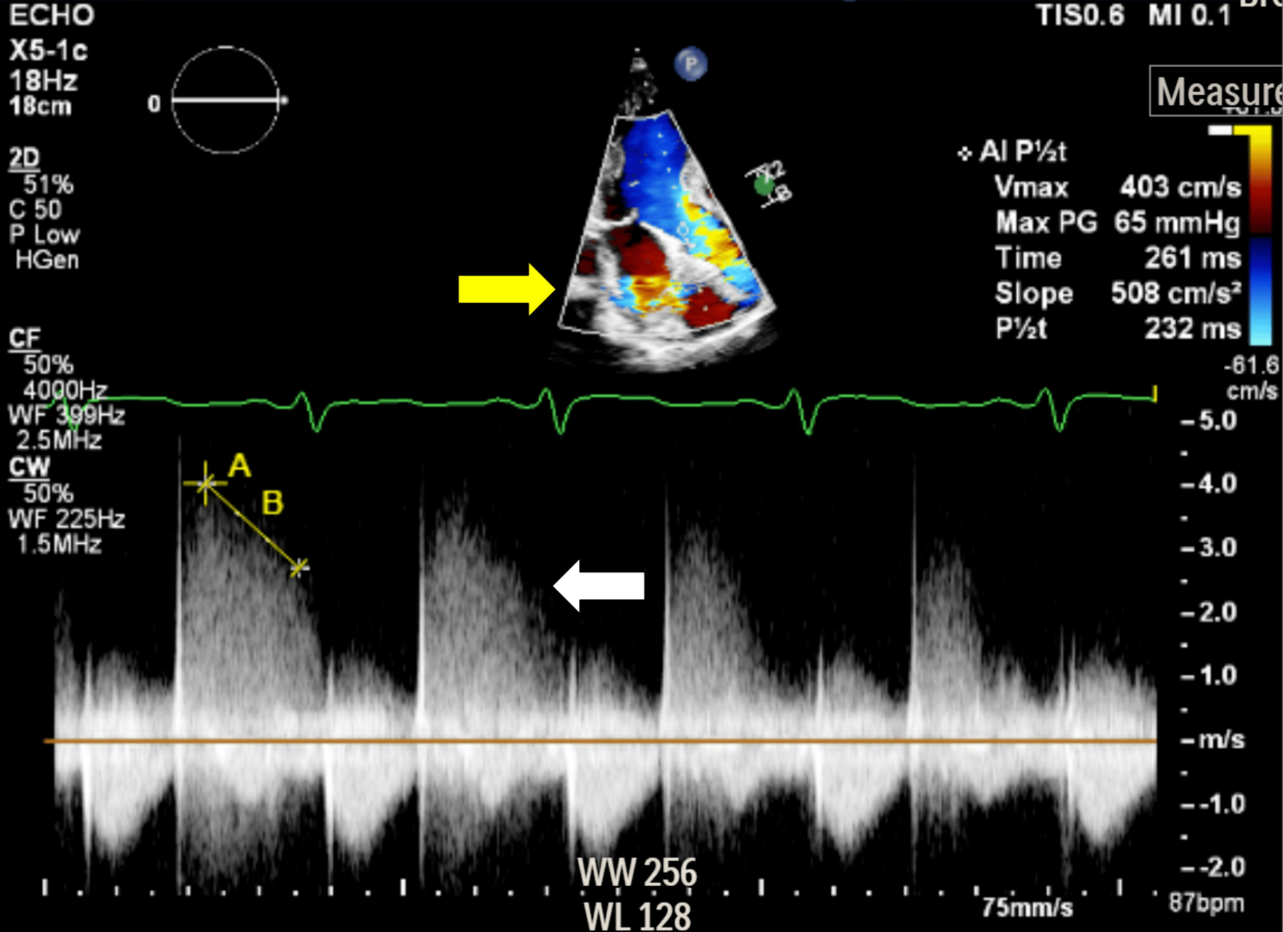
Measurement of moderate-to-severe aortic regurgitation using color flow (yellow arrow) and continuous-wave Doppler (white arrow).

A TEE was subsequently performed on hospital day six as part of a comprehensive cardiovascular evaluation. It confirmed left and right atrial dilation with an echogenic area involving the right and left coronary cusps, consistent with endocarditis (Figure 3). Color flow and continuous-wave Doppler studies demonstrated moderate-to-severe aortic regurgitation as well (Figure 4). Although one may attribute these changes to the use of different imaging modalities, the patient did begin to exhibit mild lower extremity edema at this time, consistent with potential cardiovascular insufficiency. 

The patient's venous access port was removed, and cultures from the site were negative on Gram stain, acid-fast stain, and fungal culture. Despite medical management, the patient developed worsening volume overload and valvular dysfunction, requiring further intervention by cardiothoracic surgery. Ultimately, the patient and multidisciplinary care team decided that an aortic valve replacement was the best course of action.

**Figure 5:**
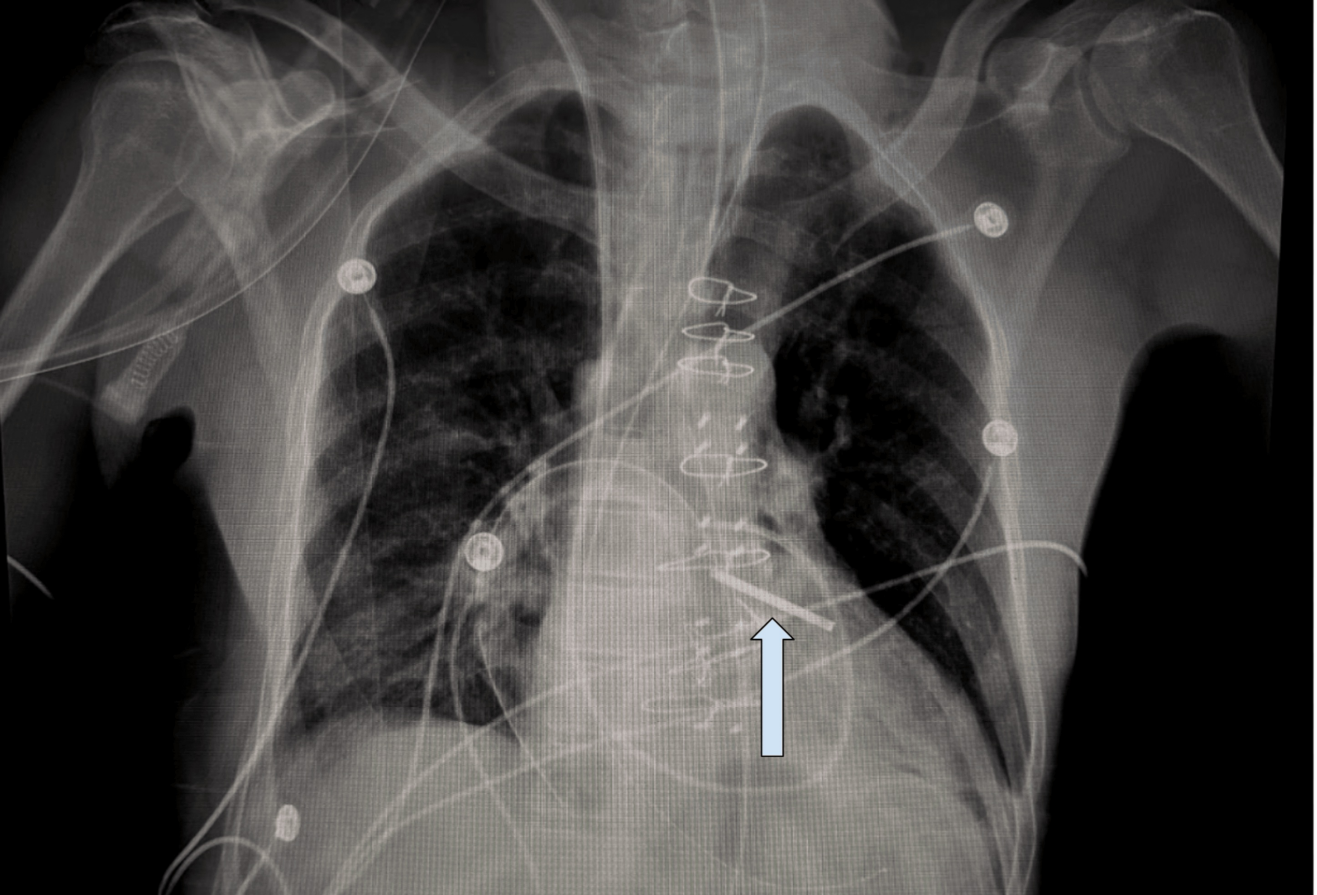
Postoperative chest X-ray depicting valve replacement and left atrial appendage clip (arrow).

The patient was scheduled for a preoperative cardiac catheterization, which ultimately depicted no coronary artery disease. Cardiothoracic surgery performed a 27 mm aortic valve replacement, debridement of the pulmonary valve due to a filamentous structure visualized intraoperatively, and insertion of a 40 mm left atrial appendage clip (Figure 5). Intraoperatively, he required substantial transfusion support consistent with sickle cell anemia and perioperative hemolysis; 1200 mL red blood cells (RBC), 600 mL leukoreduced RBC (six units total), 693 mL fresh-frozen plasma, and 500 mL platelets were cumulatively transfused. The patient tolerated the procedure well, and there were no complications. 

Post-operative recovery was notable for resolving pulmonary edema and improving thrombocytopenia, but no recurrent bacteremia. The patient was discharged on postoperative day 10 with a peripherally inserted central catheter in place, with recovery progressing appropriately. Oral discharge medications included atorvastatin 40 mg daily, deferasirox 360 mg four times a day, folic acid 1 mg daily, furosemide 40 mg daily, lisinopril 2.5 mg daily, metoprolol tartrate 12.5 mg twice daily, orphenadrine 100 mg twice daily, and oxycodone extended-release 30 mg twice daily. Intravenous medication for discharge was vancomycin 1000 mg in 0.9% sodium chloride IV infused at 166.7 mL/hr to be continued for 18 days after discharge. He also continued his regimen of crizanlizumab 5 mg/kg IV once every four weeks for sickle cell disease, which he has been on for the past five years. A conversation pertaining to venous access port reinsertion was not initiated; the plan at this time was to maintain hemoglobin above 8.0 and transfuse as needed. 

## Discussion

Aortic valve endocarditis in individuals with sickle cell disease (SCD) is infrequently reported; however, these patients remain uniquely vulnerable to severe infection and septic deterioration. Functional asplenia, chronic hemolysis, immune dysregulation, and recurrent vascular access increase susceptibility to bacteremia, while microvascular injury and chronic ulceration provide potential portals of entry for pathogens [7]. Although* Staphylococcus capitis* is traditionally regarded as a low-virulence coagulase-negative *Staphylococcus*, its pathogenic potential becomes clinically significant in the presence of indwelling vascular devices or when bacteremia persists despite appropriate antibiotic therapy [8]. This case illustrates that even low-virulence organisms may precipitate destructive valvular disease in patients with underlying hematologic vulnerability.

Sepsis-related inflammatory activation and endothelial injury likely contributed to the patient’s rapid valvular compromise [9]. The vegetation and mild-to-moderate aortic insufficiency identified on initial TTE progressed to moderate-to-severe aortic insufficiency rapidly, as demonstrated on TEE, despite targeted antimicrobial therapy. Such accelerated progression has been described in patients with hemoglobinopathies, in whom systemic inflammation, endothelial dysfunction, and vaso-occlusive stress increase tissue fragility and susceptibility to rapid decompensation [10]. These findings demonstrate the importance of serial cardiac imaging in SCD patients with persistent fever or bacteremia, even when initial echocardiographic abnormalities may not seem severe.

Management was further complicated by progressive thrombocytopenia, prompting concern for medication-related effects and requiring transition from cefazolin to vancomycin. In SCD, thrombocytopenia may reflect sepsis-associated consumption, marrow stress, or acute hemolysis, and its development complicates perioperative decision-making related to anticoagulation, transfusion strategy, and surgical timing [11]. Perioperative red blood cell exchange was therefore critical to improve oxygen delivery and mitigate intraoperative sickling during cardiopulmonary bypass [12]. This intervention remains essential for high-risk cardiac surgery in patients with SCD and likely contributed to the patient’s favorable postoperative course.

Yet another unique vulnerability has been reported in a recent case of *Candida parapsilosis* endocarditis of the native tricuspid valve in a patient with sickle cell disease, despite the absence of traditional risk factors for fungal endocarditis [13]. Although the pathogen, valve involved, and clinical manifestations differed, both cases illustrate how sickle cell-associated immune dysfunction, chronic tissue injury, and endothelial stress can permit atypical organisms to cause aggressive valvular disease. These cases suggest that in SCD, host vulnerability, rather than pathogenic virulence alone, may explain the rapid progression and clinical decompensation.

This case emphasized the need for continued vigilance for endocarditis in SCD patients presenting with sepsis, particularly when indwelling vascular devices or chronic ulcers are present. Early echocardiography, frequent reassessment of valvular function, and cardiothoracic surgical assessment are paramount. Ultimately, rapid recognition, appropriate antibiotic adjustment, and coordinated multidisciplinary planning resulted in successful valve replacement and functional recovery. 

## Conclusions

Native aortic valve endocarditis due to *Staphylococcus capitis* in sickle cell disease is rare yet capable of rapid valve destruction and hemodynamic collapse. The intersection of sickle cell disease and destructive aortic valve endocarditis is only minimally represented in available literature, highlighting a gap in clinical guidance. This case demonstrates the importance of serial echocardiography, antibiotic selection, and the vitality behind conducting a risk-benefit analysis for surgical intervention when aortic insufficiency progresses. Swift recognition and timely surgical valve replacement achieved clinical stabilization and cardiac function on postoperative assessment. 
